# Author Correction: Expected effects of a global transformation of agricultural pest management

**DOI:** 10.1038/s41467-026-71991-y

**Published:** 2026-04-13

**Authors:** Niklas Möhring, Malick N. Ba, Anna Rafaela Cavalcante Braga, Sabrina Gaba, Vesna Gagic, Per Kudsk, Ashley Larsen, Robin Mesnage, Urs Niggli, Matin Qaim, Pepijn Schreinemachers, Christian Stamm, Wim de Vries, Robert Finger

**Affiliations:** 1 Production Economics Group, University of Bonn, Bonn, Germany; 2 World Vegetable Center, Cotonou, Benin; 3 Department of Chemical Engineering, Universidade Federal de São Paulo, São Paulo, Brazil; 4 USC 1339 Centre d′Etudes Biologiques de Chizé- Résilience, INRAE CNRS La Rochelle Université, Villiers-en-Bois, France; 5 Department of Agriculture and Fisheries Queensland, Brisbane, QLD Australia; 6 Department of Agroecology, Aarhus University, Flakkebjerg, Denmark; 7 Bren School of Environmental Science & Management, UC Santa Barbara, Santa Barbara, CA USA; 8 Department of Medical & Molecular Genetics, Kings’s College London, London, UK; 9 Institute of Agroecology, Aarau, Switzerland; 10 Center for Development Research (ZEF), University of Bonn, Bonn, Germany; 11 World Vegetable Center, Bangkok, Thailand; 12 Eawag, Swiss Federal Institute of Aquatic Science and Technology, Dübendorf, Switzerland; 13 Environmental Systems Analysis Group, Wageningen University and Research, Wageningen, The Netherlands; 14 Agricultural Economics and Policy Group, ETH Zurich, Zurich, Switzerland

**Keywords:** Agriculture, Environmental impact, Sustainability

Correction to: *Nature Communications* 10.1038/s41467-025-66982-4, published online 8 December 2025

In the version of this article initially published, there was a coding error in the anonymization of the non-anonymous data for our analysis. Due to the coding error, duplicates of entries with identical geographic coordinates were introduced in the data. Correcting the error reduces the sample size from 517 to 473. We did not recognize this earlier since it happened in the anonymization step before the analysis. We have re-run all analyses and the conclusions are not affected. A point-by-point list of updates to the article is available as Supplementary Information accompanying this amendment. The HTML and PDF versions of the article have been updated.

## Supplementary information

List of edits to original article

