# Supplementary material for: Expected effects of a global transformation of agricultural pest management
Source: Nat Commun. 2025 Dec 8;16:10901. doi: 10.1038/s41467-025-66982-4 (PMC12686407; doi:10.1038/s41467-025-66982-4)
Supplement: Supplementary file 2 — Reporting Summary [file 41467_2025_66982_MOESM2_ESM.pdf]

nature portfolio

Corresponding author(s): Niklas Möhring, Robert Finger

Last updated by author(s): Oct 29, 2025

Reporting Summary

Nature Portfolio wishes to improve the reproducibility of the work that we publish. This form provides structure for consistency and transparency in reporting. For further information on Nature Portfolio policies, see our Editorial Policies and the Editorial Policy Checklist.

Statistics

For all statistical analyses, confirm that the following items are present in the figure legend, table legend, main text, or Methods section.

n/a	Confirmed	
☐	☒	The exact sample size (*n*) for each experimental group/condition, given as a discrete number and unit of measurement
☒	☐	A statement on whether measurements were taken from distinct samples or whether the same sample was measured repeatedly
☐	☒	The statistical test(s) used AND whether they are one- or two-sided<br>*Only common tests should be described solely by name; describe more complex techniques in the Methods section.*
☐	☒	A description of all covariates tested
☐	☒	A description of any assumptions or corrections, such as tests of normality and adjustment for multiple comparisons
☐	☒	A full description of the statistical parameters including central tendency (e.g. means) or other basic estimates (e.g. regression coefficient) AND variation (e.g. standard deviation) or associated estimates of uncertainty (e.g. confidence intervals)
☐	☒	For null hypothesis testing, the test statistic (e.g. *F*, *t*, *r*) with confidence intervals, effect sizes, degrees of freedom and *P* value noted<br>*Give P values as exact values whenever suitable.*
☒	☐	For Bayesian analysis, information on the choice of priors and Markov chain Monte Carlo settings
☒	☐	For hierarchical and complex designs, identification of the appropriate level for tests and full reporting of outcomes
☒	☐	Estimates of effect sizes (e.g. Cohen's *d*, Pearson's *r*), indicating how they were calculated

Our web collection on statistics for biologists contains articles on many of the points above.

Software and code

Policy information about availability of computer code

Data collection	Survey data was collected using LimeSurvey Community Edition Version 5.6.10+230313.
Data analysis	Data was prepared and analyzed using R version 4.2.1.<br>The code used for analysis is ppublicly provided - see code availability statement

For manuscripts utilizing custom algorithms or software that are central to the research but not yet described in published literature, software must be made available to editors and reviewers. We strongly encourage code deposition in a community repository (e.g. GitHub). See the Nature Portfolio guidelines for submitting code & software for further information.

Data

Policy information about availability of data

All manuscripts must include a data availability statement. This statement should provide the following information, where applicable:

- Accession codes, unique identifiers, or web links for publicly available datasets
- A description of any restrictions on data availability
- For clinical datasets or third party data, please ensure that the statement adheres to our policy

Data collected and used for the analysis is made publically available and a data and code accession statement is included in the manuscript.
Data availability:
A documentation of the complete survey text is provided in the supplementary materials of the manuscript. The data used in this study are available in the

bonndata database under accession code https://doi.org/10.60507/FK2/XWSS9W.
Code availability:
A documentation of the complete R Code used for the analysis is provided together with the manuscript. It can be used together with the freely available data (see data availability) to reproduce all analyses and figures. The code used in this study are available in the bonndata database under accession code https://doi.org/10.60507/FK2/FE09XJ.

Research involving human participants, their data, or biological material

Policy information about studies with human participants or human data. See also policy information about sex, gender (identity/presentation), and sexual orientation and race, ethnicity and racism.

Reporting on sex and gender	Findings were not differentiated by sex or gender. Sex, gender nor any other personal characteristics were considered in the study design or determined based on self-reporting or methods used. The only criterion for inclusion in the survey was expertise in the research area, independent of personal characteristics.
Reporting on race, ethnicity, or other socially relevant groupings	The only criterion for inclusion in the survey was expertise in the research area independent of personal characteristics. Only expertise-related characteristics (area of expertise, research field, strength of expertise) were used as co-variates.
Population characteristics	The only criterion for inclusion in the survey was expertise in the research area independent of personal characteristics. Only expertise-related characteristics (area of expertise, research field, strength of expertise) were used as co-variates.
Recruitment	The population of experts for sustainable pest management is limited and we divided it in expertise by region and field of research. In order to contact i) only experts in sustainable pest management, ii) a diversity of experts, iii) with sufficient sample sizes for each field of expertise and region we used a three-tiered recruitment strategy: a.) Snowball system based on the networks of co-authors; b.) Academic experts through literature databases; and c.) Members of leading international organisations in the area of pest management. We discuss advantages and disadvantages in terms of selection bias for each strategy in detail in the manuscript and how they are complementary in alleviating these. We further test for potential differences in results from different recruitment strategies and find no impacts (see manuscript).
Ethics oversight	The survey was assessed and approved by the ethics council of ETH Zurich before its distribution (approval number 2022-N-34).

Note that full information on the approval of the study protocol must also be provided in the manuscript.

Field-specific reporting

Please select the one below that is the best fit for your research. If you are not sure, read the appropriate sections before making your selection.

☐ Life sciences ☒ Behavioural & social sciences ☐ Ecological, evolutionary & environmental sciences

For a reference copy of the document with all sections, see nature.com/documents/nr-reporting-summary-flat.pdf

Behavioural & social sciences study design

All studies must disclose on these points even when the disclosure is negative.

Study description	Data used are quantitative. Data come from a global survey, conducted for the study, merged with external data sources on a region- and country-level. See manuscript for details.
Research sample	The targeted population are leading global experts in sustainable pest management. The exact population size is unknown. Through our three-tier sampling strategy we are confident to have captured an important part of this population in our survey. The criteria for selection of participants was expertise on sustainable pest management in different geographic zones and research disciplines. We aimed to at least have 30 respondents from each of these sub-categories and achieved this goal accept for Oceania. Detailed statistics and discussions on the sample and its distribution are provided in the manuscript. Findings were not differentiated by sex or gender. Sex, gender nor any other personal characteristics were considered in the study design or determined based on self-reporting or methods used. The only criterion for inclusion in the survey was expertise in the research area, independent of personal characteristics. We merged the survey data with external data on regional development (Human Development Index, Planetary pressure-adjusted Human Development Index (United Nations), GDP per capita (World Bank)), progress on associated SDGs (Our World in Data) and characteristics of the farming system (potential pest damages (Oerke, 2006), attainable crop yields (Our World in Data), agricultural productivity (output value per hectare, (FAO)), and current pesticide pollution (Tang et al., 2021). External data come from publically available databases (as indicated in brackets above). For detailed explanations see the manuscript.
Sampling strategy	The population of experts for sustainable pest management is limited and we divided it in expertise by region and field of research. In order to contact i) only experts in sustainable pest management, ii) a diversity of experts, iii) with sufficient sample sizes for each field of expertise and region we used a three-tiered recruitment strategy: a.) Snowball system based on the networks of co-authors; b.) Academic experts through literature databases; and c.) Members of leading international organisations in the area of pest management. We discuss advantages and disadvantages in terms of selection bias for each strategy in detail in the manuscript and how they are complementary in alleviating these. We further test for potential differences in results from different recruitment strategies and find no impacts (see manuscript). Survey data was collected using an online survey in LimeSurvey Community Edition

	Version 5.6.10+230313. In the survey we further highlight the importance of collecting a diversity of opinions from different disciplines. As the population of leading experts from sustainable pest management is unknown but expected to be limited (we identify 1531 corresponding authors in tier 2 of the sampling strategy), we targeted a sample size of at least n=30 per subcategory (geographic region and research field) as a reasonable compromise. This was reached, except for the region of Oceania. We are confident that our total sample of 517 complete responses captures an important part of leading experts. See manuscript for a detailed discussion.
Data collection	The survey was conducted online using the LimeSurvey software. The survey design was verified in several steps (see manuscript for details).
Timing	The survey was sent out with two reminders for non-respondents respectively. It was open from the end of March 2022 – October 2022.
Data exclusions	All complete responses were used for the analysis. Please see the manuscript for a detailed description.
Non-participation	For the three tiers of the sampling strategy, a definitive response rate to the survey can only be defined for tier 2 (Academic experts through literature databases). In this tier the response rate (complete responses) was 15.6% (240 out of 1531).
Randomization	Participants were not allocated in random groups. In the analysis, we controlled for co-variates regarding geographic region of expertise, research field, strength of expertise, scope of expertise, expected importance of sub-field for the research question.

Reporting for specific materials, systems and methods

We require information from authors about some types of materials, experimental systems and methods used in many studies. Here, indicate whether each material, system or method listed is relevant to your study. If you are not sure if a list item applies to your research, read the appropriate section before selecting a response.

Materials & experimental systems

n/a	Involved in the study
☒	☐ Antibodies
☒	☐ Eukaryotic cell lines
☒	☐ Palaeontology and archaeology
☒	☐ Animals and other organisms
☒	☐ Clinical data
☒	☐ Dual use research of concern
☒	☐ Plants

Methods

n/a	Involved in the study
☒	☐ ChIP-seq
☒	☐ Flow cytometry
☒	☐ MRI-based neuroimaging

Plants

Seed stocks	NA
Novel plant genotypes	NA
Authentication	NA
