# Supplementary material for: Author Correction: Expected effects of a global transformation of agricultural pest management
Source: Nat Commun. 2026 Apr 13;17:3446. doi: 10.1038/s41467-026-71991-y (PMC13076632; doi:10.1038/s41467-026-71991-y)
Supplement: Supplementary file 1 — List of edits to original article [file 41467_2026_71991_MOESM1_ESM.pdf]

Detailed point-by-point corrections for Möhring et al. (2025)

Manuscript text

**Page, line and section**	**Correction**
Introduction	
p. 2, l.31	The indicated sample size needs to be changed from 517 to 473.
p. 5, l. 111	The indicated sample size needs to be changed from 517 to 473.
Results	
p. 6, l.170	The share of respondents with at least one negative response needs to be changed from 45 to 43%
p.6, l.172	The share of negative responses for ECON2 needs to be changed from 28 to 26%
p. 8, ll.210-211	The range of correlation values needs to be changed from |0.01-0.27| to |0.00-0.25|
p. 15 ll.253-254	The point estimate needs to be changed from 0.19 to 0.15 and the respective value in brackets with CI from −0.19 [−0.33; −0.06] to −0.15 [−0.29; −0.01].
p. 15, l. 256	The coefficient estimate and CI in brackets needs to be changed from 0.16 [−0.02; 0.33] to 0.15 [−0.04; 0.33].
p. 16 l.256	The coefficient estimate and CI in brackets needs to be changed from−0.38 [−0.72; −0.04] to −0.42 [−0.79; −0.06].
p.16 ll. 266-267	The coefficient estimate and CI in brackets needs to be changed from −0.49 [−0.88; −0.11] to −0.48 [−0.89; −0.06]
p. 16, l.268	The coefficient estimate and CI in brackets needs to be changed from 0.77 [0.25; 1.30] to 0.74 [0.18; 1.30].
p. 17, ll.294-296	The point estimate needs to be changed from 0.1 to 0.09.
p. 17, ll.317-325	In the sentence "For all regions and production systems, we consistently find that "identifying and providing access to effective and cost-efficient substitutes for pesticides" (i.e., accounting for prices and efficiency in protecting crops) is the topmost priority for experts to support the transformation (22/100 points, SD = 0.14) followed by "education and extension on pest management" (18/100 points, SD = 0.13), "economic support" (13/100 points, SD = 0.11), "legislative support" (12/100 points, SD = 0.10) and "awareness building" (i.e., of unintended effects) (12/100 points, SD = 0.10) as the top five responses (see supplementary fig. 8 for an overview of all responses)."<br>- The mean needs to be changed to 22/100 from21/100<br>- The standard deviations need to be changed as follows, "identifying and providing access to effective and cost-efficient substitutes for pesticides" (SD = 0.14), "education and extension on pest management" (SD = 0.13), "economic support" (SD = 0.11), "legislative support" (SD = 0.10) and "awareness building" (SD = 0.10).
**Methods**	
p. 30, l.696	The indicated sample size needs to be changed from 517 to 473.
p. 30, l.707	The indicated sample size needs to be changed from 517 to 473.

Manuscript Figures and Tables

**Figure/Table**	**Comparison/Correction**
Figure 1 was updated; the updated file is in the clean word file and attached as a separate file	All conclusions remain the same; No visible changes; Underlying mean per indicator only changed by 2% on average.

	Figure Legend: The indicated sample size needs to be changed from 517 to 473.
Figure 2 was updated; the updated file is in the clean word file and attached as a separate file	All conclusions remain the same; No visible changes; Underlying mean per indicator only changed by 2% on average.<br><br>Figure Legend: The indicated sample size needs to be changed from 166 to 128 for Europe, 75 to 73 for Africa and 94 to 90 for Asia.
Figure 3 was updated; the updated file is in the clean word file and attached as a separate file	All conclusions remain the same; No changes in direction of coefficients; Minor changes in significance of individual coefficients.
Table 2 was updated; the updated file is in the clean word file and attached as a separate file	All conclusions remain the same; All summary statistics were updated; almost no change in absolute values: means of the 24 indicator variables only deviate by 2% on average.<br><br>Table headline: The indicated sample size needs to be changed from 517 to 473.

Supplementary materials

Page, line and section	Correction
Supplementary Note 2	
p. 4	"We contacted them to respond to the survey and asked them to additionally forward the survey to senior members of their organizations in other regions worldwide and received 199 complete responses."<br><br>The indicated sample size needs to be changed from 223 to 199.
p. 4	"Out of the 1531 identified corresponding authors, we received 274 complete responses, amounting to a response rate of 17.9%."<br><br>The indicated sample size needs to be changed from 294 to 274 and the response rate from 19.3 to 17.9%.
Supplementary Note 4	
p. 9	"Overall, 84 out of 473 respondents (17.8%) gave comments, with a broad distribution across disciplines and expert regions."<br><br>The indicated sample size needs to be changed from 517 to 473.<br>The share of respondents needs to be changed from 16.2 to 17.8%.
p.10-11	The shares need to be changed from 1.) 6.8 to 7.4%, 2.) 5.4 to 5.9%, 3.) 2.3 to 2.5% and 4.) 1.2 to 1.3%.

Figure/Table	Comparison/Correction
Supplementary Table 1 was updated in the clean supplementary materials file	All conclusions remain the same; Most CVs remain exactly the same; Minor absolute changes between |0.01|-|0.03| in individual CV values.
Supplementary Table 2 was updated in the clean supplementary materials file	All conclusions remain the same; No notable changes in sign, magnitude or dimension of correlations; Minor absolute changes between |0.01|-|0.05| in individual correlation values.
Supplementary Table 3 was updated in the clean supplementary materials file	All conclusions remain the same; No change in direction of coefficients; Minor changes in significance of individual coefficients.
Supplementary Table 4 was updated in the clean supplementary materials file	All conclusions remain the same; No changes in relative distributions of answers.
Supplementary Fig. 1 was updated in the clean supplementary materials file	All conclusions remain the same; No visible changes; See Table 2 updated for descriptive statistics.<br><br>Figure Legend: The indicated sample size needs to be changed from 517 to 473.
Supplementary Fig. 3 was updated in the clean supplementary materials file	All conclusions remain the same; No changes in shapes of distribution; Lower sample size leads to lower density.<br><br>Figure Legend: The indicated sample size needs to be changed from 517 to 473. The shares of negative responses need to be changed to: 43% at least gave one negative response across all

	indicators they assessed. The share of negative responses across all responses and per indicator was: ECON1 = 0.20, ECON2 = 0.26, ECON3 = 0.05, ECON4 = 0.16, ECON5 = 0.10, ECON6 = 0.02, ENV1 = 0.03, ENV2 = 0.03, ENV3 = 0.06, ENV4 = 0.02, ENV5 = 0.03, FS1 = 0.20, FS2 = 0.09, FS3 = 0.05, FS4 = 0.15, HH1 = 0.02, HH2 = 0.03, HH3 = 0.04, HH4 = 0.03, SOC1 = 0.08, SOC2 = 0.13, SOC3 = 0.11, SOC4 = 0.11, SOC5 = 0.03.
Supplementary Fig. 4 was updated in the clean supplementary materials file	All conclusions remain the same; No visible changes.<br><br>Figure Legend: The indicated sample size needs to be changed from 517 to 473.
Supplementary Fig. 5 was updated in the clean supplementary materials file	All conclusions remain the same; No visible changes.<br><br>Figure Legend: The indicated sample size needs to be changed from 517 to 473.
Supplementary Fig. 6 was updated in the clean supplementary materials file	All conclusions remain the same; No visible changes.<br><br>Figure Legend: The indicated sample size needs to be changed from 517 to 473.
Supplementary Fig. 7 was updated in the clean supplementary materials file	All conclusions remain the same; No visible changes.<br><br>Figure Legend: The indicated sample size needs to be changed from 517 to 473.
Supplementary Fig. 8 was updated in the clean supplementary materials file	All conclusions remain the same; No changes in significant differences/equality between measures.<br><br>Figure Legend: The indicated sample size needs to be changed from 517 to 473.
Supplementary Fig. 9 was updated in the clean supplementary materials file	All conclusions remain the same; Distribution between continents even more equal now; Figure shows that the overwhelming majority of duplicates where in Europe, which is already oversampled.<br><br>Figure Legend: The indicated sample size needs to be changed from 514 to 470.
Supplementary Fig. 10 was updated in the clean supplementary materials file	All conclusions remain the same; No visible change in relative distributions.<br><br>Figure Legend: The indicated sample size needs to be changed from 517 to 473.
Supplementary Fig. 11 was updated in the clean supplementary materials file	All conclusions remain the same; No visible change in relative distributions.

	Figure Legend: The indicated sample size needs to be changed from 517 to 473.
Supplementary Fig. 12 was updated in the clean supplementary materials file	All conclusions remain the same; No visible change in relative distributions.<br><br>Figure Legend: The indicated sample size needs to be changed from 517 to 473.
Supplementary Fig. 13 was updated in the clean supplementary materials file	All conclusions remain the same; No change in direction of coefficients; Minor changes in significance of individual coefficients.
Supplementary Fig. 14 was updated in the clean supplementary materials file	All conclusions remain the same; No visible change in relative distribution.<br><br>Figure Legend: The indicated sample size needs to be changed from 517 to 473.
